# Successful multimodal treatment of intraoral salivary duct carcinoma in a patient with multiple lymph node metastases: a case report

**DOI:** 10.1186/s12957-016-1090-3

**Published:** 2017-01-10

**Authors:** Shuichi Imaue, Kei Tomihara, Takeru Hamashima, Gakuto Tomizawa, Kuninori Nomura, Masakiyo Sasahara, Makoto Noguchi

**Affiliations:** 1 Department of Oral and Maxillofacial Surgery, Graduate School of Medicine and Pharmaceutical Sciences for Research, University of Toyama, 2630 Sugitani, Toyama city, Toyama 930-0194 Japan; 2 Department of Pathology, Graduate School of Medicine and Pharmaceutical Sciences for Research, University of Toyama, 2630 Sugitani, Toyama city, Toyama 930-0194 Japan; 3 Department of Radiology, Graduate School of Medicine and Pharmaceutical Sciences for Research, University of Toyama, 2630 Sugitani, Toyama city, Toyama 930-0194 Japan

**Keywords:** Lymph node metastasis, Multimodal therapy, Salivary duct carcinoma

## Abstract

**Background:**

Salivary duct carcinoma (SDC) is a high-grade salivary gland malignancy that is associated with an aggressive clinical behavior and poor prognosis. Herein, we report on a long surviving case of SDC of the minor salivary gland with multiple lymph node metastases (LNMs).

**Case presentation:**

An 83-year-old woman presented with a history of lymphadenopathy in the right side of the neck and recent onset and rapid growth of a mass in the right buccal region. Clinical examinations and biopsy findings were suggestive of a salivary gland malignant tumor with regional LNMs. The patient was treated with neoadjuvant chemotherapy. Tumor excision and ipsilateral radical neck dissection were performed, followed by adjuvant chemoradiotherapy. Postoperative histological examination revealed a tumor with irregular nests of atypical ductal epithelial cells, a cribriform growth pattern, and comedo-like central necrosis that lead to a final diagnosis of SDC. LNMs were observed in six lymph nodes of the right side of the neck. The patient underwent postoperative chemotherapy using single-agent cisplatin that was administered concurrently with radiotherapy (total, 65 Gy). There was no evidence of local recurrence or distant metastasis for >6 years.

**Conclusions:**

Although available data on treatment modalities for SDC remain limited, multimodal therapy may contribute to improved clinical outcomes in patients with advanced intraoral SDC.

## Background

Salivary duct carcinoma (SDC) is a rare malignant tumor that arises from the ductal epithelial cells of the salivary glands. SDC was first described by Kleinsasser et al. [1] in 1968 as a highly aggressive, malignant salivary gland tumor. It was then classified as a distinct entity of salivary gland tumors by the World Health Organization in 1991 [2]. SDC most frequently arises in the major salivary glands, especially the parotid glands. However, it can also occur more infrequently in the minor salivary glands of the oral cavity [3]. SDC has an aggressive clinical behavior and poor clinical outcome that is characterized by the rapid growth of the disease, multiple nodal metastases, early distant metastasis (DM), and a high rate of recurrence [3]. Recently, prognostic factors for SDC have been extensively studied in large numbers of patients and it has been suggested that adjuvant therapy may improve the clinical outcome of patients with advanced SDC [3–8]. However, because the incidence of SDC of minor salivary gland origin is very low compared to that of SDC of major salivary gland origin, and a limited number of cases have been reported to date, the clinical outcome and benefit of adjuvant therapy for SDC of minor salivary gland origin remains to be elucidated. Therefore, additional studies are required to better understand the prognostic factors for patients with intraoral SDC.

To the best of our knowledge, we are the first to report on the treatment outcome of a long surviving intraoral SDC patient with multiple lymph node metastases (LNMs).

## Case presentation

**Fig. 1:**
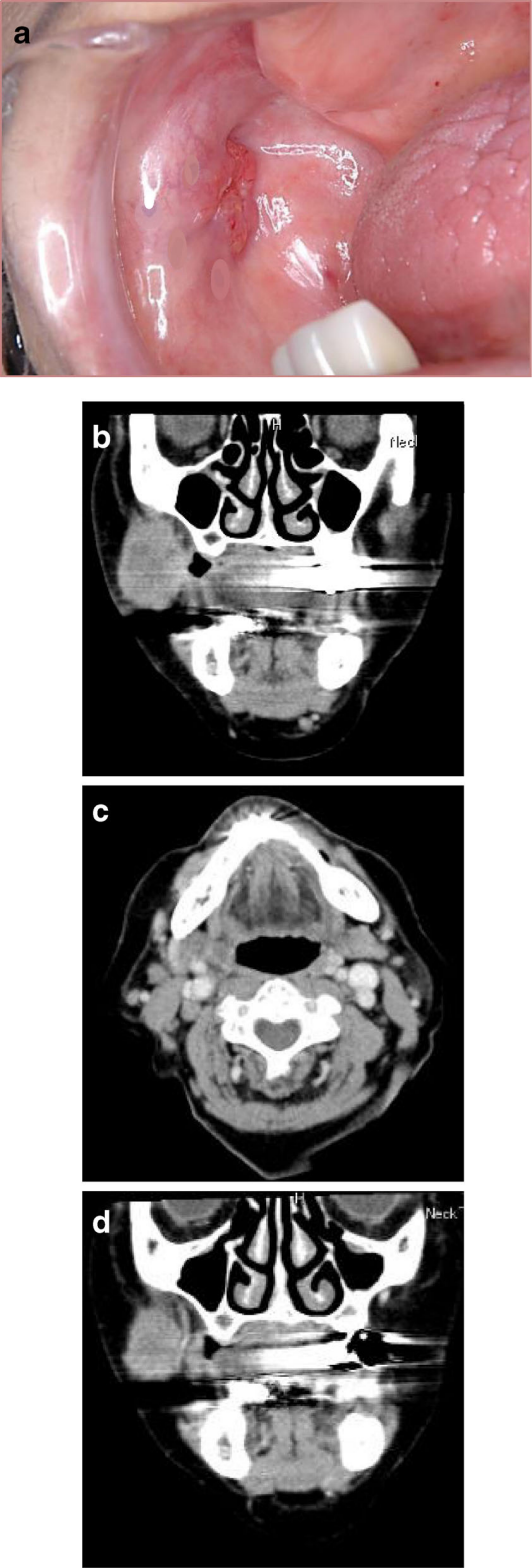
**a** An intraoral photograph revealing an ulcerated lesion in the right buccal region. **b**, **c** Computed tomography demonstrating a homogeneously enhanced lesion in the right buccal region and multiple, enlarged lymph nodes in the right side of the neck. The largest diameter of the tumor on initial assessment (**b**) and after induction chemotherapy (**d**) was 34.2 to 26.0 cm, respectively. The tumor exhibited a reduction in size (**d**) in response to induction chemotherapy using an intra-arterial infusion of high-dose cisplatin and concurrent peroral TS-1

An 83-year-old woman, complaining of an asymptomatic painless swelling in the right buccal region, was referred to our department in 2009. The lesion had not been long standing and had recently gradually increased in size. Initially, the patient was seen by a general dental practitioner, when she had first noticed the lesion, and was subsequently referred to the Department of Oral and Maxillofacial Surgery at a local hospital where a diagnosis of a neoplasm of the buccal region was suggested. She was then referred to our department. The patient had a medical history of myocardial infarction that was treated with coronary artery stenting at the age of 76 years. On initial assessment, no systemic symptoms were evident. Extraoral and intraoral examination revealed a lesion in the right buccal region, measuring 2.0 × 1.8 cm, that was palpable, indurated, and elastically hard without trismus (Fig. 1a). The overlying mucosa was partially ulcerated. Laboratory examinations revealed no significant findings except for elevated levels (6.3 ng/mL) of tumor marker serum cytokeratin fragment 21.1. Squamous cell carcinoma and carcinoembryonic antigen levels were 2.1 and 2.4 ng/mL, respectively. Computed tomography exhibited homogeneous enhancement of an ill-demarcated lesion in the right buccal region (Fig. 1b). Computed tomography of the neck detected several enlarged lymph nodes in ipsilateral levels I–III that suggested the presence of LNMs (Fig. 1c). No other specific findings were observed by computed tomography of the abdominal or thoracic regions. An intraoral biopsy was performed and histopathological findings were suggestive of a malignant salivary gland neoplasm. Accordingly, the tumor in the right buccal region was classified as T4aN2bM0.

Initially, the patient was treated with induction chemotherapy using an intra-arterial infusion of high-dose cisplatin (100 mg/body on day 1) and concurrent peroral TS-1 (100 mg/body on days 1–14). The tumor size was reduced from 34.2 to 26.0 mm, with a tumor reduction rate of 24.0% by computed tomography measurements (Fig. 1d). Tumor excision using both intraoral and extraoral approaches and ipsilateral radical neck dissection were performed. The surgical defect of the right buccal region was patched using a split thickness skin graft.

**Fig. 2:**
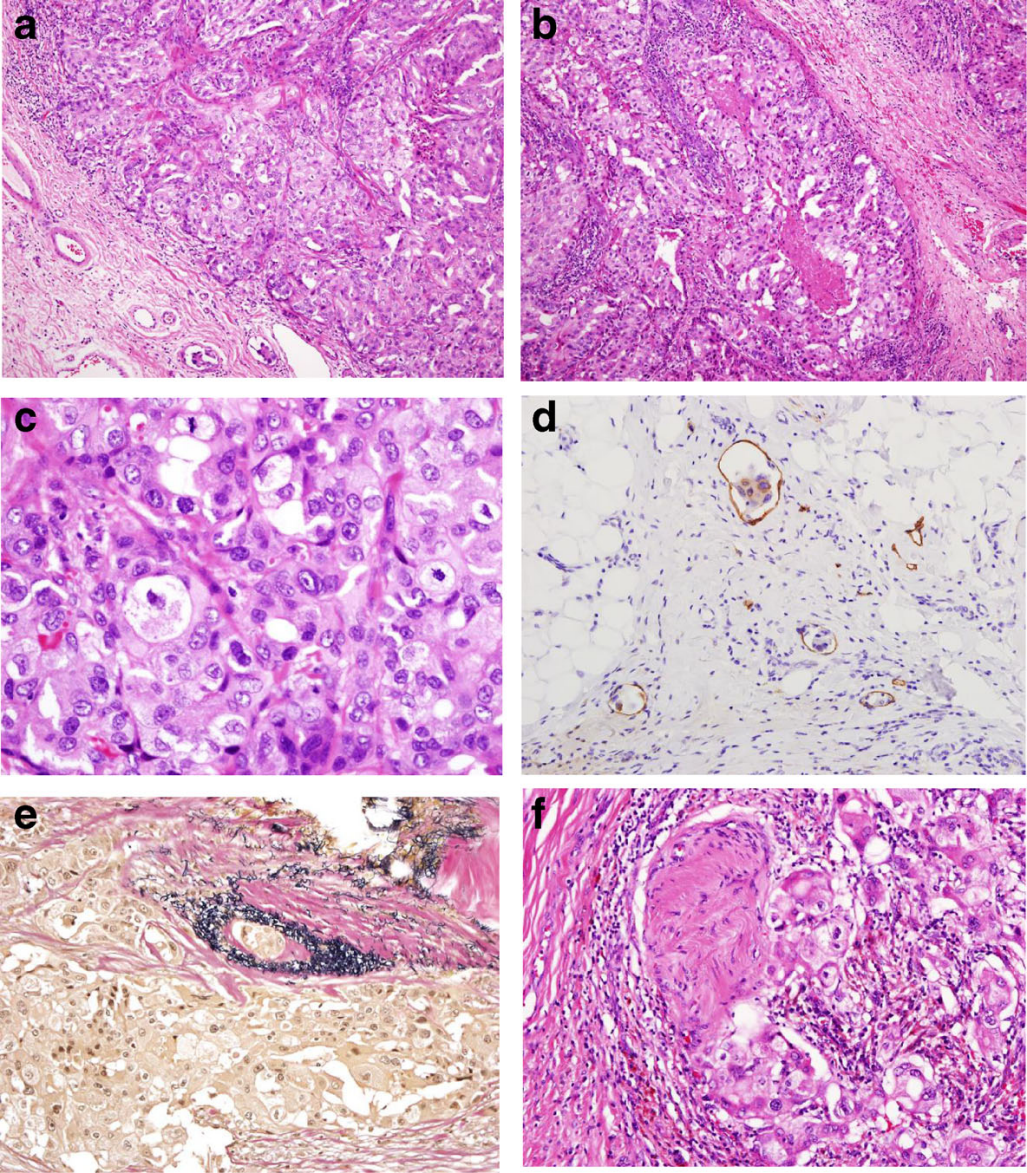
Microscopic features of salivary duct carcinoma on hematoxylin and eosin staining. **a**, **b** The tumor was composed of a neoplastic component characterized by atypical ductal epithelial cells, a cribriform growth pattern, and comedo-like central necrosis. **c** The epithelium was primarily composed of polygonal shaped cells with an abundant eosinophilic cytoplasm (numerous mitotic figures) and large nuclei (nuclear atypia). **d** Lymphatic invasion, **e** vascular invasion, and **f** perineural invasion were observed on podoplanin, elastic Van Gieson, and hematoxylin and eosin staining, respectively

**Fig. 3:**
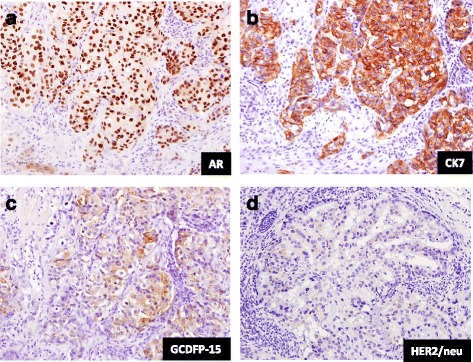
Immunohistochemical staining for androgen receptor, cytokeratin 7, gross cystic disease fluid protein-15 (GCDFP-15), and human epithelial growth factor receptor 2 (HER2)/neu. The tumor cells were immunoreactive for **a** androgen receptor and **b** cytokeratin 7 and focally immunoreactive for **c** GCDFP-15 but were not immunoreactive for (**d**) HER2/neu

Macroscopically, the tumor measured 2.2 × 2.2 × 1.7 cm. It had a solid, whitish appearance with muscle layer invasion at the cut surface. The overlying mucosal surface was partially ulcerated. Microscopically, the lesion was composed of a neoplastic component characterized by atypical ductal epithelial cells, a cribriform growth pattern, and comedo-like central necrosis (Fig. 2a, b). The epithelium was primarily composed of polygonal shaped cells with an abundant eosinophilic cytoplasm and large nuclei (Fig. 2c). The tumor was distinct from the parotid gland, suggesting that it had originated from the minor salivary gland of the buccal region. Lymphovascular invasion and perineural invasion (PNI) were observed (Fig. 2d–f). Furthermore, LNMs were evident in six (level IB [*n* = 1], IIA [*n* = 2], and IIB [*n* = 3]) of the 25 dissected lymph nodes. Metastatic lymph nodes exhibited similar histopathology findings as the primary site of the lesion. The tumor cells stained positive for androgen receptor, epithelial membrane antigen (EMA), cytokeratin 7, and gross cystic disease fluid protein-15 (GCDFP-15) but negative for alpha-smooth muscle actin (α-SMA), calponin, carcinoembryonic antigen (CEA), cytokeratin 14, human epidermal growth factor receptor 2 (HER2)/neu, estrogen receptor, S100 protein, vimentin, and tumor protein p53 (Fig. 3a–d). The Ki-67 proliferation index was 36.0%. According to these histopathological features, the tumor was diagnosed as a SDC of minor salivary gland origin. One month after surgery, the patient was treated with chemotherapy using single-agent cisplatin (5 mg/m [2]) that was administered concurrently with radiotherapy (total, 65 Gy) for the primary lesion and ipsilateral neck. The patient has remained alive for >6 years after the initial diagnosis with no evidence of recurrence.

### Discussion

SDC exhibits an aggressive clinical behavior and especially poor prognosis that is characterized by multiple nodal metastases, early DM, and a high rate of local recurrence [3, 4]. According to reports in the literature [3], the rates of nodal metastasis, DM, and local recurrence are approximately 60, 50, and 50%, respectively [3]. Moreover, the mean overall survival was 56 months and the 5-year disease-free survival rates for stage I–IV SDC were approximately 42, 40, 30, and 23%, respectively [3].

Recently, prognostic factors for SDC have been extensively studied in large numbers of patients and several factors, including tumor size, anatomical location, age, positive infiltrative margin, PNI, regional recurrence, nodal metastasis, and DM have been suggested to be associated with a poor prognosis [3, 4, 9, 10]. In particular, it has been suggested that a large tumor size of >3 cm is associated with a poor prognosis [11], while a small tumor size of <2 cm is associated with a more favorable prognosis [9]. However, the prognostic data described above were established based on the findings of SDC of major salivary gland origin, owing to the fact that the majority of SDC cases arise in the major salivary glands, particularly the parotid glands. Therefore, prognostic data concerning SDC of minor salivary gland origin remains limited, because the incidence of SDC of minor salivary gland origin is extremely low compared to that of SDC of major salivary gland origin. A relatively favorable prognosis in patients with SDC of minor salivary gland origin compared to SDC of parotid gland origin was suggested based on the finding that the former was associated with less frequent regional LNMs in a review of the literature [10, 12]. However, this less aggressive behavioral tendency of SDC of minor salivary gland origin is most likely due to the relatively smaller tumor size, because SDCs of minor salivary gland origin are detected earlier than SDCs of major salivary gland origin [10]. There have been no reports describing the difference in prognosis between patients with SDC of minor salivary gland origin and patients with SDC of major salivary gland origin after matching for stage. Histologically, SDC resembles high-grade ductal carcinoma of the breast with a solid or cribriform growth pattern. The majority of immunohistochemical staining investigations [1–3] have revealed similarities between SDC and breast carcinoma with positive immunostaining for epithelial markers such as cytokeratin and epithelial membrane antigen and particularly intense immunoreactivity for gross cystic disease fluid protein-15 (GCDFP-15), androgen receptor, and HER2/neu. It has been suggested that overexpression of HER2/neu and tumor protein p53 are associated with a poor clinical course, including early regional recurrences, DM, and low survival rates [3]. Although no consistent therapeutic concept exists for this entity, complete surgical resection with radical neck dissection, followed by radiotherapy, has been suggested as the treatment recommendation for resectable SDC [13, 14]. Postoperative radiotherapy has been suggested to improve locoregional control in a patient with advanced stage SDC [13]. In particular, observations of PNI during a final pathological examination may lead to consideration of postoperative radiotherapy [13]. Moreover, only limited data are available regarding the efficacy of chemotherapeutic agents. Therefore, no consensus exists for the efficacy of chemotherapy. Molecularly targeted therapy against HER2 protein with anti-HER2 monoclonal antibodies has been suggested to be contributive as a therapeutic target for patients with HER2/neu-positive SDC [15, 16]. Anti-androgen therapy has also been evaluated in patients with androgen receptor positive SDC [17].

In the present case, multiple regional LNMs and an elevated Ki-67 proliferation index were observed. Lymphovascular invasion and PNI were also apparent on histological examination. Therefore, multimodal therapy with induction chemotherapy, radical surgery, and postoperative adjuvant chemoradiotherapy was conducted. In fact, in the present case, induction chemotherapy with cisplatin led to a 24.0% reduction in tumor size, suggesting that chemotherapy is at least partially effective in treating patients with SDC. To the best of our knowledge, this is the first report describing the efficacy of multimodal therapy for SDC of minor salivary gland origin.

Additional studies are required to elucidate further the clinical outcomes of patients with SDC of minor salivary gland origin. Particular attention should be paid to the potential application of multimodal adjuvant therapy, especially when endeavoring to accumulate adequate experience of this rare type of tumor. Taking into account the aggressive clinical behavior of SDC, such as the high locoregional recurrence rate and early DM, multimodal therapy should be considered for the management of this high-grade malignancy.

## Conclusions

We present a rare case of SDC of minor salivary gland origin that was successfully treated with radical surgery followed by adjuvant chemotherapy and radiotherapy despite the presence of multiple regional LNMs. Particular attention was paid to the potential application of multimodal adjuvant therapy, especially when endeavoring to accumulate adequate experience of this rare type of tumor.

## Abbreviations

α-SMA: Alpha-smooth muscle actin
CEA: Carcinoembryonic antigen
DM: Distant metastasis
EMA: Epithelial membrane antigen
GCDFP-15: Gross cystic disease fluid protein-15
HER2: Human epithelial growth factor receptor 2
LNM: Lymph node metastasis
PNI: Perineural invasion
SDC: Salivary duct carcinoma

## References

[1] Kleinsasser O, Klein HJ, Hübner G (1968). Salivary duct carcinoma. A group of salivary gland tumors analogous to mammary duct carcinoma. Arch Klin Exp Ohren Nasen Kehlkopfheilkd.

[2] Seifert G, Sobin LH (1991). Histological typing of salivary gland tumours, 2nd ed.

[3] Jaehne M, Roeser K, Jaekel T, Schepers JD, Albert N, Löning T (2005). Clinical and immunohistologic typing of salivary duct carcinoma: a report of 50 cases. Cancer.

[4] Jayaprakash V, Merzianu M, Warren GW (2014). Survival rates and prognostic factors for infiltrating salivary duct carcinoma: analysis of 228 cases from the Surveillance, Epidemiology, and End results database. Head Neck.

[5] Otsuka K, Imanishi Y, Tada Y (2016). Clinical outcomes and prognostic factors for salivary duct carcinoma: a multi-institutional analysis of 141 patients. Ann Surg Oncol.

[6] Wee DT, Thomas AA, Bradley PJ (2012). Salivary duct carcinoma: what is already known, and can we improve survival?. J Laryngol Otol.

[7] Shinoto M, Shioyama Y, Nakamura K (2013). Postoperative radiotherapy in patients with salivary duct carcinoma: clinical outcomes and prognostic factors. J Radiat Res.

[8] Nakashima T, Yasumatsu R, Toh S (2015). Is there a role of adjuvant treatment for salivary duct carcinoma?. J Laryngol Otol.

[9] Pons Y, Alves A, Clément P, Conessa C (2011). Salivary duct carcinoma of the parotid. Eur Ann Otorhinolaryngol Head Neck Dis.

[10] Urban SD, Hall JM, Bentkover SH, Kadish SP (2002). Salivary duct carcinoma of minor salivary gland origin: report of a case involving the cavernous sinus. J Oral Maxillofac Surg.

[11] Brandwein MS, Jagirdar J, Patil J, Biller H, Kaneko M (1990). Salivary duct carcinoma (cribriform salivary carcinoma of excretory ducts). A clinicopathologic and immunohistochemical study of 12 cases. Cancer.

[12] Huh KH, Heo MS, Lee SS, Choi SC (2003). Three new cases of salivary duct carcinoma in the palate: a radiologic investigation and review of the literature. Oral Surg Oral Med Oral Pathol Oral Radiol Endod.

[13] Guzzo M, Locati LD, Prott FJ, Gatta G, McGurk M, Licitra L (2010). Major and minor salivary gland tumors. Crit Rev Oncol Hematol.

[14] Simpson RH (2013). Salivary duct carcinoma: new developments—morphological variants including pure in situ high grade lesions; proposed molecular classification. Head Neck Pathol.

[15] Prat A, Parera M, Reyes V (2008). Successful treatment of pulmonary metastatic salivary ductal carcinoma with trastuzumab-based therapy. Head Neck.

[16] Kaidar-Person O, Billan S, Kuten A (2012). Targeted therapy with trastuzumab for advanced salivary ductal carcinoma: case report and literature review. Med Oncol.

[17] Jaspers HC, Verbist BM, Schoffelen R (2011). Androgen receptor-positive salivary duct carcinoma: a disease entity with promising new treatment options. J Clin Oncol.

